# Disease-Associated Mutations Disrupt Functionally Important Regions of Intrinsic Protein Disorder

**DOI:** 10.1371/journal.pcbi.1002709

**Published:** 2012-10-04

**Authors:** Vladimir Vacic, Phineus R. L. Markwick, Christopher J. Oldfield, Xiaoyue Zhao, Chad Haynes, Vladimir N. Uversky, Lilia M. Iakoucheva

**Affiliations:** 1 Department of Computer Science, Columbia University, New York, New York, United States of America; 2 Department of Chemistry and Biochemistry, University of California San Diego, La Jolla, California, United States of America; 3 Howard Hughes Medical Institute, Chevy Chase, Maryland, United States of America; 4 Center for Computational Biology and Bioinformatics, Department of Biochemistry and Molecular Biology, Indiana University School of Medicine, Indianapolis, Indiana, United States of America; 5 Cold Spring Harbor Laboratory, Cold Spring Harbor, New York, United States of America; 6 Laboratory of Statistical Genetics, The Rockefeller University, New York, New York, United States of America; 7 Department of Molecular Medicine, University of South Florida College of Medicine, Tampa, Florida, United States of America; 8 Institute for Biological Instrumentation, Russian Academy of Sciences, Pushchino, Moscow Region, Russia; 9 Department of Psychiatry, University of California San Diego, La Jolla, California, United States of America; Bar Ilan University, Israel

## Abstract

The effects of disease mutations on protein structure and function have been extensively investigated, and many predictors of the functional impact of single amino acid substitutions are publicly available. The majority of these predictors are based on protein structure and evolutionary conservation, following the assumption that disease mutations predominantly affect folded and conserved protein regions. However, the prevalence of the intrinsically disordered proteins (IDPs) and regions (IDRs) in the human proteome together with their lack of fixed structure and low sequence conservation raise a question about the impact of disease mutations in IDRs. Here, we investigate annotated missense disease mutations and show that 21.7% of them are located within such intrinsically disordered regions. We further demonstrate that 20% of disease mutations in IDRs cause local disorder-to-order transitions, which represents a 1.7–2.7 fold increase compared to annotated polymorphisms and neutral evolutionary substitutions, respectively. Secondary structure predictions show elevated rates of transition from helices and strands into loops and *vice versa* in the disease mutations dataset. Disease disorder-to-order mutations also influence predicted molecular recognition features (MoRFs) more often than the control mutations. The repertoire of disorder-to-order transition mutations is limited, with five most frequent mutations (R→W, R→C, E→K, R→H, R→Q) collectively accounting for 44% of all deleterious disorder-to-order transitions. As a proof of concept, we performed accelerated molecular dynamics simulations on a deleterious disorder-to-order transition mutation of tumor protein p63 and, in agreement with our predictions, observed an increased α-helical propensity of the region harboring the mutation. Our findings highlight the importance of mutations in IDRs and refine the traditional structure-centric view of disease mutations. The results of this study offer a new perspective on the role of mutations in disease, with implications for improving predictors of the functional impact of missense mutations.

## Author Summary

Intrinsically unstructured or disordered proteins have been implicated in the etiology of a wide spectrum of diseases. However, the molecular mechanisms that relate mutations in intrinsically disordered regions (IDRs) to disease pathogenesis have not been investigated. Disordered proteins do not conform to the prevailing view of deleterious mutations which equates function, structure and evolutionary conservation – intrinsically disordered regions are functional, but lack a fixed three-dimensional structure and in general have low sequence conservation. Here we demonstrate that >20% of disease-associated missense mutations affect IDRs and interfere with their functions. We further show that 20% of deleterious mutations in IDRs induce predicted disorder-to-order transitions. Our predictions are supported by accelerated molecular dynamics simulations that show an increase in helical propensity of the region harboring a disease disorder-to-order transition mutation of tumor protein p63. Our results refine the traditional structure-centric view of disease mutations and offer a new perspective on the role of non-synonymous mutations in disease. Our findings have broad implications for improving predictors of the functional impact of missense mutations, and for interpretation of novel variants identified in large genome sequencing projects that aim to provide a better understanding of human genetic variation and its relevance to common diseases.

## Introduction

Recent years have seen significant advancements in cataloging the genetic variation in humans and relating it to disease susceptibility. In particular, missense mutations, which introduce changes in the amino acid sequence of proteins, have been the subject of considerable attention due to the large number of ongoing exome sequencing studies. As a result, numerous computational models that classify amino acid substitutions as damaging or benign are currently available (reviewed in [1], [2], [3]). The majority of these methods rely on the information from solved or modeled protein structures [4], [5], [6], [7], [8], [9] and/or are based on evolutionary conservation, following the assumption that functionally important residues of proteins are conserved [10], [11], [12], [13]. This choice of features limits the usefulness of current methods for classifying mutations in proteins that lack a fixed structure or have low sequence conservation, both of which are hallmarks of the intrinsically disordered proteins (IDPs). Underestimating the impact of missense mutations in intrinsically disordered regions (IDRs) leads to a decrease in overall sensitivity of the existing methods. For example, it has recently been observed that SIFT predictions have more false negatives on annotated disease mutations in disordered, solvent accessible and non-conserved regions [14].

Intrinsically disordered proteins were first identified as a distinct class of proteins more than a decade ago [15], [16], [17], [18]. It has since been clearly demonstrated that IDPs are prevalent in eukaryotic proteomes [19], are involved in signaling and regulation [20], [21], carry sites of posttranslational modifications [22], [23], and serve as hubs in protein interaction networks [24], [25], [26]. Despite their important functional roles [27], [28], [29], [30], [31], IDRs generally have low sequence conservation [32], with the exception of IDRs involved in chaperone activity and RNA binding [33]. IDPs have been implicated in many human diseases, including cancer, diabetes, cardiovascular and neurodegenerative disorders [20], [34]. Due to their signaling and regulatory roles, IDPs tend to be tightly regulated, and disruptions in regulation of IDPs have been linked to disease [35]. Despite the functional importance and disease relevance of IDPs, the prevalence of disease-associated missense mutations in disordered regions and their impact on disordered conformations have not been investigated so far.

Here, we offer a new perspective on disease mutations that accounts for mutations in disordered regions. We investigate disease-associated mutations located in ordered and disordered regions, and compare them to missense mutations from two control datasets, single amino acid polymorphisms and neutral evolutionary substitutions. We demonstrate that deleterious missense mutations may affect disordered regions, thereby disrupting the disorder-based type of structure. Our results suggest that disease mutations in ordered regions (ORs) and IDRs differ substantially in frequency, properties, and functional impact. We find that disease mutations in disordered regions more frequently cause predicted disorder-to-order transitions and influence predicted disordered binding regions (MoRFs) compared to mutations from the control datasets. IDR mutations are also enriched in DNA-binding and transmembrane domains, and in sites of posttranslational modifications. Accelerated molecular dynamics simulations performed on a deleterious disorder-to-order transition mutation that affects the DNA-binding domain of tumor protein p63 support our disorder predictions. We further show that two widely used predictors of functional impact of single nucleotide variants, PolyPhen-2 and SIFT, exhibit a >10% decrease in sensitivity when predicting the effect of annotated disease mutations located in IDRs compared to ORs mutations. Our findings have broad implications for improving predictors of the functional impact of missense mutations and therefore may significantly influence the interpretation of novel variants identified in large genome sequencing projects.

## Results

### Mutation frequencies in ordered and disordered regions

We examined the frequency of annotated disease mutations (DM) from the UniProt database in predicted ordered and disordered regions and compared them to the distributions of putatively functionally neutral mutations from two control datasets, annotated polymorphisms from UniProt (Poly) and neutral evolutionary substitutions (NES) (**Materials and Methods**). We observed that disease mutations preferentially affect ordered regions, with 78.3% of them mapped to the predicted ordered regions and 21.7% mapped to the predicted disordered regions (**Table 1**). Neutral evolutionary substitutions are more evenly distributed, with 55.3% observed in ORs and 44.7% in IDRs (**Table 1**). The annotated polymorphisms show somewhat intermediate distribution, with 59.6% in ORs and 40.4% in IDRs. Enrichment of disease mutations in ordered regions agrees with previous observations that disease mutations frequently affect protein structure, activity and stability [4], [7]. Our results were consistent across three disorder predictors, VLXT [36], VSL2B [37] and IUPRED [38] (**Table S1**).

**Table 1:** Disease mutations have higher frequencies in ordered regions.

			IDR		OR		
Dataset	Proteins	Mutations	n	%	n	%	Fold
DM	2,194	15,459	3,356	21.7	12,103	78.3	-
Poly	8,489	24,220	9,790	40.4	14,430	59.6	0.54
NES	1,998	60,299	26,927	44.7	33,372	55.3	0.49

The enrichment of disease mutations in ORs cannot be explained by the overall lower disorder content of the proteins containing these mutations. Although proteins that carry disease-associated mutations are on average slightly less disordered than proteins from the Poly dataset (mean±SD 32.7±17.9% *vs* 35.3±19.5%, respectively; also see **Figure S1**), this difference is not sufficient to explain the 3.6 fold enrichment of disease mutations in ORs. Furthermore, despite the fact that the NES dataset was constructed from the same set of proteins as DM (**Materials and Methods**), only a 1.2 fold enrichment of mutations in ORs compared to IDRs is observed in this dataset (**Table 1**), which lends further support to enrichment of disease mutations in ORs. Finally, we compared mutation rates (number of amino acid changes per ordered and per disordered residue) in ORs and IDRs in all three datasets, and only in the DM dataset the mutation rate in ORs was higher than the mutation rate in IDRs (**Table 2**).

**Table 2:** Disorder-to-order transition mutations are significantly enriched in disease.

	D→O		D→D				O→D		O→O			
Dataset	n	%	n	%	Fold	*P*-value	n	%	n	%	Fold	*P*-value
DM	670	20.0	2,686	80.0	-	-	590	4.9	11,513	95.1	-	-
Poly	1,125	11.5	8,665	88.5	1.7	1.06×10^−32^	710	4.9	13,720	95.1	1.0	0.89
NES	1,971	7.3	24,956	92.7	2.7	5.47×10^−105^	1,870	5.6	31,502	94.4	0.9	0.0023

*P*-values were calculated using Fisher's exact test between mutation counts in DM and Poly or NES.

Despite the prevalence of disease mutations in ordered regions, 21.7% of DMs are mapped to the predicted disordered regions. We have investigated these mutations in greater detail, as discussed below, and mutations in IDRs form the main focus of the remainder of this study.

### Disorder-to-order (D→O) and order-to-disorder (O→D) transition mutations

Based on the predicted disorder probability score, a residue can be classified as ordered or disordered depending on whether its score is below or above a threshold of 0.5. When analyzed from an order/disorder perspective, any missense mutation can have two different outcomes: (i) it can change the prediction score sufficiently to cross the 0.5 threshold, which would result in a conversion of the prediction from disorder to order, or from order to disorder; or (ii) it can preserve the order/disorder assignment. Thus, the effect of missense mutations can be classified as D***→***D (disorder-to-disorder) or O***→***O (order-to-order) when disorder and order assignments do not change; and as D***→***O (disorder-to-order) or O***→***D (order-to-disorder) transitions when predicted disorder and order classes switch.

Disease mutations mapped to disordered regions cause D***→***O transitions significantly more frequently than neutral evolutionary substitutions or polymorphisms (**Table 2**). We observed that 20% of the disease mutations in disordered regions result in a D***→***O transition, compared to only 11.5% and 7.3% in the Poly and NES control sets (Fisher's exact *P* = 1.06·10^−32^ and 5.47·10^−105^, respectively). In contrast, the rates of O***→***D transition show no change or a slight depletion in DM compared to Poly and NES, respectively (**Table 2**). Similar results were obtained using three different disorder predictors (**Table S3**). These observations suggest that disease mutations in disordered regions are more likely to cause a significant structural perturbation, and possibly disrupt functions that necessitate protein disorder. Below, we examine the structural and functional implications of disease mutations in greater detail.

### Secondary structure predictions and mutations

**Figure 1:**
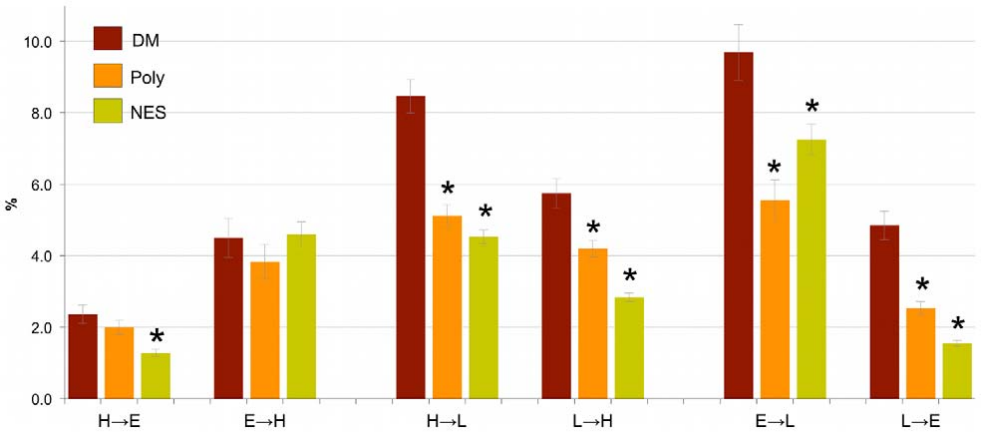
Transitions between helices (H), strands (E) and loops (L) upon mutations from three datasets, based on PHDsec predictions. Transitions from helix or strand to loop and *vice versa* are significantly enriched in disease. *P*-values were calculated using Fisher's exact test between mutation counts in DM *vs* Poly or NES. Categories with Bonferroni-corrected *P*-values<0.05 are marked with an asterisk.

To better understand how disease mutations influence protein secondary structure, we applied the secondary structure predictor PHD [39] to both the disease and control datasets. In each dataset, we calculated the frequencies of secondary structure elements (helices, strands and loops) and transitions between them upon a mutation. Overall, we observed that disease mutations affect helices and strands more frequently than control mutations (**Table S4**). We also observed that although most mutations do not cause a change in the assignment to a predicted helix, strand or loop, there is nevertheless a statistically significant increase in transitions between secondary structure elements caused by disease mutations compared to the control datasets (**Table S5**). This increase was most pronounced for transitions from helices and strands into loops, and to a lesser extent for transitions from loops into helices and strands (**Figure 1**). There was no significant difference between disease and control mutations for transitions from helix into strand and *vice versa* (**Figure 1**). Although similar trends are observed for loops predicted by PHD and disordered regions predicted by VLXT, VSL2B and IUPred (see **Figure 1**, **Table 2** and **Table S3**), it is important to note that predicted regions of disorder and loops do not necessarily overlap [40], [41], and that many secondary structure elements predicted by PHD are found within experimentally verified disordered regions [40], [42], [43].

Despite the lack of stable secondary and tertiary structure in disordered regions, the dynamic behavior of IDRs does not preclude formation of short transient secondary structure elements. These short transient elements, or Molecular Recognition Features (MoRFs) [44], frequently mediate interactions of IDRs with their physiological binding partners [44], [45], [46]. Below, we investigated the influence of missense mutations on MoRFs.

### Disease mutations in predicted α-MoRF regions

Molecular recognition features (MoRFs) are short order-prone segments within longer disordered regions that fold upon binding to their interaction partners [47]. α-MoRFs specifically form α-helices upon binding. We predicted the presence of α-MoRFs at the position of the residue both before and after it was mutated, and classified the mutation as falling into one of the three categories: (i) “predicted MoRF lost” - an α-MoRF was predicted to overlap the position of the mutated residue in the wild-type sequence but not in the mutant sequence; (ii) “predicted MoRF gained” - an α-MoRF was predicted not to overlap the position of the mutated residues in the wild-type sequence but was predicted to overlap the position of the mutated residue in the mutant sequence; (iii) “MoRF present, no change” - an α-MoRF was predicted to overlap the mutated position in both the wild-type and the mutant sequences. Mutations where an α-MoRF was absent from both wild-type and mutant sequences were not taken into account. Amino acid substitutions were placed into IDR and OR categories based on the wild-type disorder score. Details of MoRF predictions are provided in the **Materials and Methods** and in the **Supplementary Text S1**.

**Figure 2:**
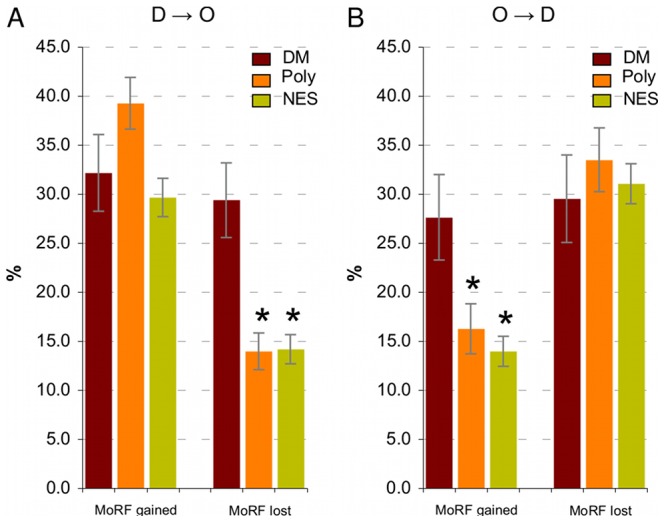
Disease D→O transition mutations lead to the loss of predicted α-MoRFs (panel A), while O→D transition mutations lead to the gain of predicted α-MoRFs (Panel B) significantly more frequently than control mutations. Y-axes show fractions of all D→O and O→D mutations that cause loss or gain of the predicted α-MoRFs, and error bars correspond to one standard deviation.

IDR mutations lead to gain or loss of predicted α-MoRFs 2.2 to 5.1 times more frequently than OR mutations, independent of the dataset used (**Figure S2**). Disease mutations in IDRs lead to a loss of predicted α-MoRFs 1.39 times more frequently than Poly and 1.36 times more frequently than NES (Fisher's exact *P* = 0.0012 and 7.9·10^−4^, respectively). Disease mutations in ORs have an opposite effect – they lead to a gain of predicted α-MoRFs 1.5-fold more frequently than Poly and 1.8-fold more frequently than NES (*P* = 0.0020 and 1.65·10^−5^). A follow up investigation showed that D***→***O and O→D mutations significantly contribute to the observed effect (**Figure 2**). Disease D***→***O mutations lead to a loss of predicted α-MoRFs 2.1-fold more frequently than Poly and NES (*P* = 1.11·10^−4^ and 5.68·10^−5^), and similarly disease O→D mutations lead to a gain of predicted α-MoRFs 1.7-fold more frequently than Poly and 2.0-fold more frequently than NES (*P* = 0.025 and 0.0012).

### Disease mutations in eukaryotic linear motifs (ELMs)

We also examined the influence of disease and control mutations on Eukaryotic Linear Motifs (ELMs), short (3 to 11 residues) conserved sequence motifs that play roles in mediating cell signaling, controlling protein turnover and directing protein localization [48]. ELMs were previously shown to be enriched in IDRs [49]. We mapped mutations from the three datasets onto 1040 annotated ELM instances from human proteome (see http://elm.eu.org/elms/browse_instances.html) and found that only 99 mutations overlap an ELM. Although disease D***→***O mutations were slightly enriched in ELMs in comparison to control D***→***O mutations (**Table S6**), this difference reached statistical significance only for DM *vs* NES (P = 0.012), but not for DM *vs* Poly (P = 0.22), likely due to a limited number of observations. We did not observe any differences for other classes of mutations. Although a decisive conclusion about enrichment of D***→***O disease mutations within ELMs could not be made at this point, we believe that the trend towards such enrichment warrants further investigation when larger numbers of ELMs and annotated mutations become available.

### Functional characterization of disease mutations in IDRs and ORs

**Figure 3:**
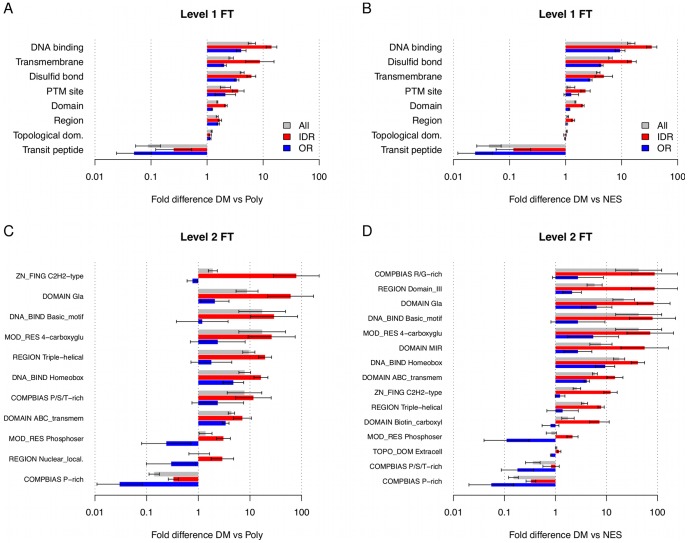
Fold differences of UniProt FT annotations in DM compared to Poly (panels A,C) and NES (panels B,D) that show statistically significant frequency difference between mutations in IDR and OR. Top row (A, B) contains level 1 and the bottom row (C, D) level 2 features. Error bars are one standard error of fold difference. Categories are sorted by decreasing fold difference in DM compared to controls.

To characterize the functional impact of missense mutations, we examined UniProt region/residue feature annotations associated with each mutation (**Materials and Methods**). A number of functional annotations for disease mutations in IDRs and ORs show significant differences in fold enrichment (**Figure 3**). Disease mutations in disordered regions are enriched in domains and functions associated with DNA binding motifs (homeobox, zinc finger, basic motif), transmembrane domains, sites of post-translational modifications, disulfide bond formation, and triple helical regions, which are often found in cytoskeletal and coiled-coil proteins. Some of these functional categories were previously strongly associated with disordered regions [28], [30], and many DNA-binding domains are known to be either entirely or partially disordered when not associated with DNA [50], [51], [52]. Further investigation of keywords associated with D***→***O transitions shows an enrichment of functions similar to IDR, while O→D transition mutations show enrichment in ABC transporter and ATP-binding regions (**Tables S7** and **S8**).

### D→O and O→D mutation patterns are different

**Figure 4:**
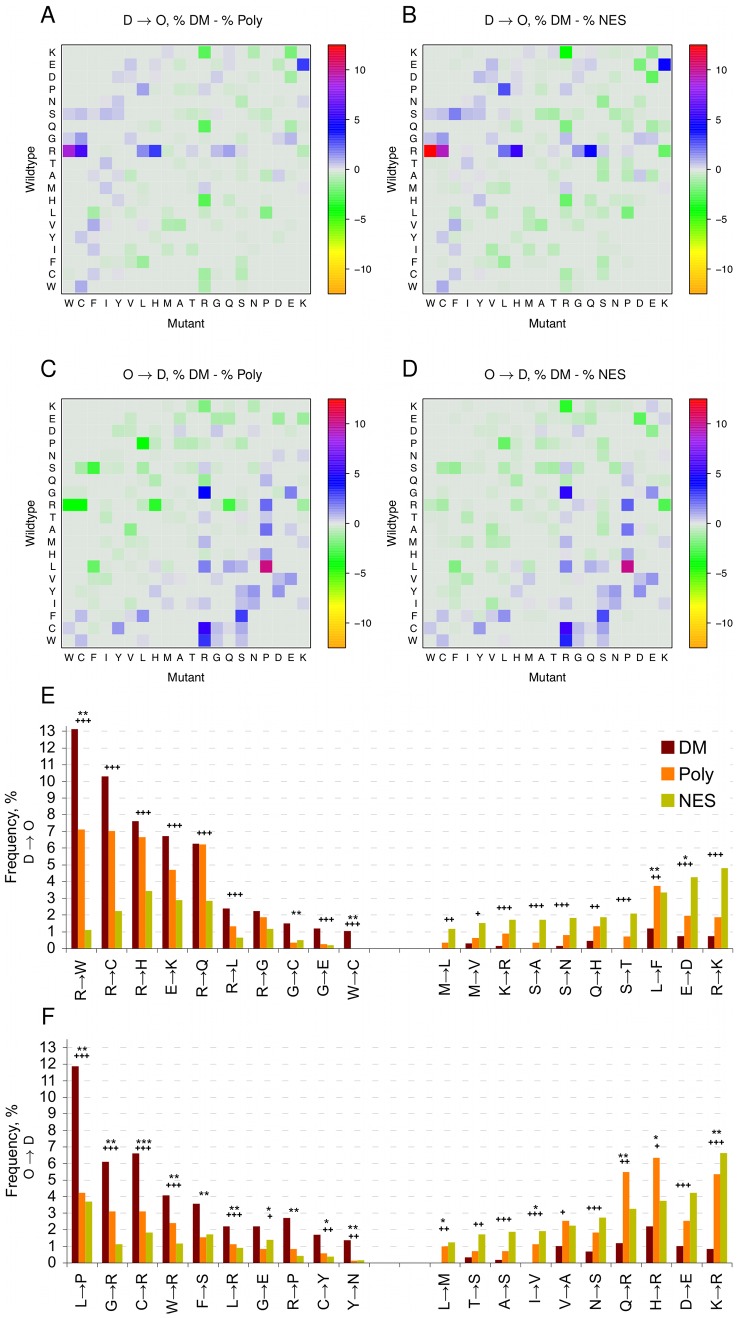
Enrichment and depletion of D*→*O (panels A, B, E) and O*→*D (panels C, D, F) mutations as % of disease mutations in comparison to % of polymorphisms (Poly; panels A, C) and % of neutral evolutionary substitutions (NES; panels B, D). In the heatplots (panels A, B, C, D) wild-type residues are on the Y-axes and the mutant residues on the X-axes. The residues are arranged according to the Vihinen flexibility scale [83]. In panels E and F, frequencies of top ten D→O (panel E) and O→D (panel F) mutations enriched and depleted in the DM dataset are shown. * signify Fisher's exact *P*-values of DM *vs.* Poly; + signify p-values of DM *vs.* NES. *** or +++ − *P*<0.001; ** or ++ − *P* between 0.001–0.01; * or + − *P* between 0.01–0.05.

In order to investigate mutations that contribute to the observed D***→***O and O***→***D transitions, we calculated the “wild-type residue→mutant residue” transition matrices in all three datasets and compared the differences in frequencies of D***→***O (**Figure 4**, first row) and O***→***D (**Figure 4**, second row) mutations between DM and Poly (**Figures 4A and 4C**), and DM and NES (**Figures 4B and 4D**). We observe that certain residue-into-residue substitutions are enriched (red), while others are depleted (green) in disease. Arginine (R) is the most frequently mutated residue in the D***→***O dataset, and leucine (L) is most frequently mutated in the O***→***D dataset. The overall results do not depend on the choice of the control dataset (Poly or NES).

The heat plots in **Figure 4** point to specific mutations that are highly enriched in disease. The most frequent disease mutation that causes a disorder-to-order transition is R→W (**Figure 4C**). Other D***→***O transition mutations significantly enriched in the DM dataset include most notably R→C, R→H, E→K, R→Q (**Figure 4E**, left section). Several other types of disorder-to-order transition mutations, such as R→K, E→D, L→F, S→T, are significantly depleted in the DM dataset (**Figure 4E**, right section), which demonstrates that distinct types of mutations preferentially occur within disease and control categories.

To verify that this result is not an artifact of our analysis, for example due to general enrichment of R→W mutations in disordered regions, or the choice of control datasets, we have compared the frequencies of R→W substitutions from this study to the matrices constructed based on the alignments of completely disordered sequences [53]. This comparison showed that in general R→W substitutions occur extremely rarely in disordered regions (with 0.11% in D85 matrix and 0.03% in D40 matrix), whereas we find R→W substitution with much higher frequency in our datasets (11.69% in DM, 6.52% in Poly, and 0.95% in NES). This result suggests that the R→W mutation is truly enriched among disease mutations.

Another category of amino-acid substitutions in DM, albeit not significantly enriched as a group, involve order-to-disorder mutations, such as L→P, C→R, G→R, W→R and others (**Figure 4F**). Some of the enriched order-to-disorder mutations are inverses of the enriched disorder-to-order mutations, such as W→R, C→R, L→R, whereas some are shared between D→O and O→D, such as G→E. This shared category points to the fact that there is no strong preference for glycine and glutamic acid to be located in either ordered or disordered regions, as reflected by the presence of both residues in the middle of the TOP-IDP scale of residue disorder propensities [54].

In summary, our analysis shows that a limited set of mutations accounts for a large fraction of all D→O and O→D transitions in the DM dataset. The top five disorder-to-order transition mutations (R→W, R→C, E→K, R→H and R→Q) collectively account for 44.0% of all D→O disease mutations, and the top five order-to-disorder transition mutations (L→P, C→R, G→R, W→R and F→S) collectively account for 32.2% of all O→D disease mutations (**Figure 4C and 4F**). Specific knowledge of the mutations responsible for such transitions may help the development of new classifiers to better predict the effects of mutations in IDRs.

### Arginine is the most frequently mutated residue in DM

We next compared the frequencies of wild-type and mutant residues in all datasets to the frequencies of typical human proteins from the UniProt database (**Figure S3**). Mutations of arginine and glycine are most dominant in DM and account for 28.5% of all disease mutations, 18.6% of all Poly and only 11.1% of NES mutations (**Figure S3B**). After normalizing by the baseline residue frequency [55] (**Figure S3A**), mutations of cysteine and tryptophane stood out, reflecting that in DM these two resides are mutated significantly above what is expected based on their frequency of occurrence in the human proteome. Interestingly, tryptophane and cysteine, and to a lesser degree histidine, are the residues into which other residues most frequently mutate, with a more pronounced effect in IDRs than in ORs (**Figure S4**).

High mutability of arginine, also observed in earlier studies [56], [57], together with the **high propensity of arginine mutations to cause disorder-to-order transitions** suggest an underlying mechanism which predisposes arginine to be a frequent target for disease mutations. Arginine is encoded by 6 distinct codons, 4 of which contain the CG dinucleotide (CGG, CGT, CGC and CGA). DNA methylation often involves CpG dinucleotides and due to spontaneous deamination 5-methylcytosine is more prone to mutating into T. Upon a C-to-T transition, the first three arginine codons would become codons for W (TGG) or C (TGT, TGC), and the last one would create a stop codon (TGA). The observed high frequency of R→W and R→C in DM and low frequency in control datasets (**Figure S5**) argues in favor of negative selection against these amino acid substitutions, which frequently cause predicted disorder-to-order transitions in proteins.

### Mutations in IDRs are less accurately predicted

**Figure 5:**
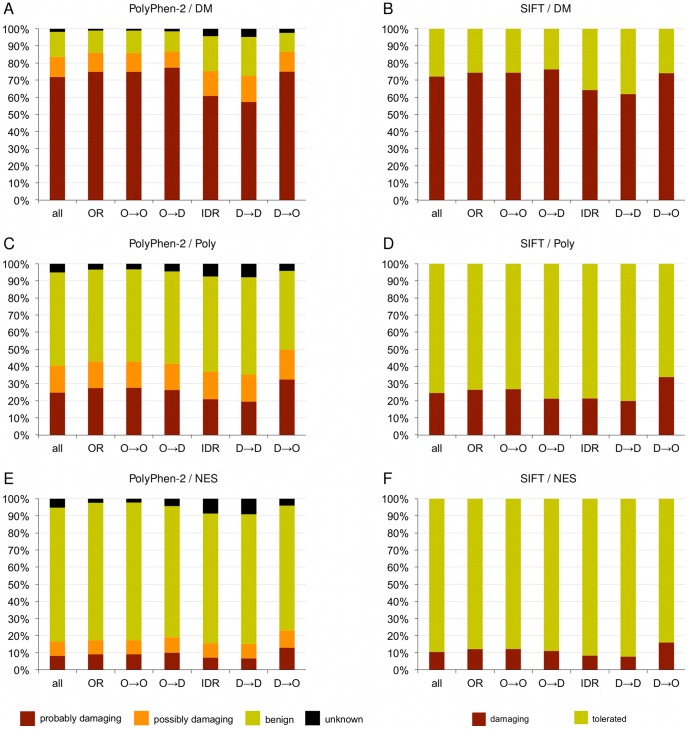
Distribution of PolyPhen-2 (panels A, C, E) and SIFT (panels B, D, F) calls for mutations in DM, Poly and NES datasets. Both predictors show a drop in sensitivity for disease mutations in IDR and D***→***D categories (A, B) and a drop in specificity for D***→***O mutations in Poly (C, D) and NES (E, F).

A recent study demonstrated that SIFT has a higher error rate when predicting the impact of SNVs in solvent accessible and disordered protein regions [14]. In order to rigorously evaluate this statement, SIFT [10] and PolyPhen-2 [58] were applied to all mutations in DM, Poly and NES datasets, and the prediction accuracies on mutations in different order/disorder categories were compared (**Figure 5** and **Table S9**). Both SIFT and PolyPhen-2 predict significantly less disease mutations as deleterious in IDRs than in ORs (SIFT “damaging” 64.3% *vs* 74.4%, χ^2^ *P* = 4.19·10^−28^; PolyPhen-2 “probably damaging” 60.8% *vs* 74.9%, *P* = 8.05·10^−74^). SIFT and PolyPhen-2 both predict significantly more polymorphisms to be benign in IDRs than in ORs (SIFT “tolerated” 78.7% *vs* 73.5%, *P* = 1.86 ·10^−18^; PolyPhen-2 “benign” 55.5% *vs* 53.7%, *P* = 3.74 ·10^−64^), and likewise for neutral evolutionary substitutions (SIFT 91.6% *vs* 87.9%, *P* = 7.38·10^−45^, PolyPhen-2 80.2% *vs* 75.6%, *P* = 2.84·10^−215^). IDR mutations seem to be more difficult to handle for the PolyPhen-2 model in general, and in all three datasets more IDR than OR mutations are returned as “unknown” (DM 4.4% *vs* 1.2%, Poly 7.5% *vs* 3.4%, NES 8.7% *vs* 2.4%). Upon closer examination, we determined that among DM mutations, the D→D transition category was the most difficult to predict correctly for both predictors, while the D→O category was most often correctly predicted as deleterious. However, in the case of D→O mutations, higher sensitivity comes at the expense of lower specificity, and significantly more mutations from Poly and NES are predicted as deleterious in D→O transitions than in any other category (**Table S9**). Similar results were obtained by analyzing raw PolyPhen-2 and SIFT scores (**Figures S6** and **S7**). Notably, the DM dataset investigated here overlaps with the predictors' training sets, and the reported accuracies are likely to be lower when applied to out-of-training set examples. In summary, our findings underscore the need for incorporating features of IDRs into predictive disease mutation models.

### Accelerated molecular dynamics simulations of p63 D→O mutation

**Figure 6:**
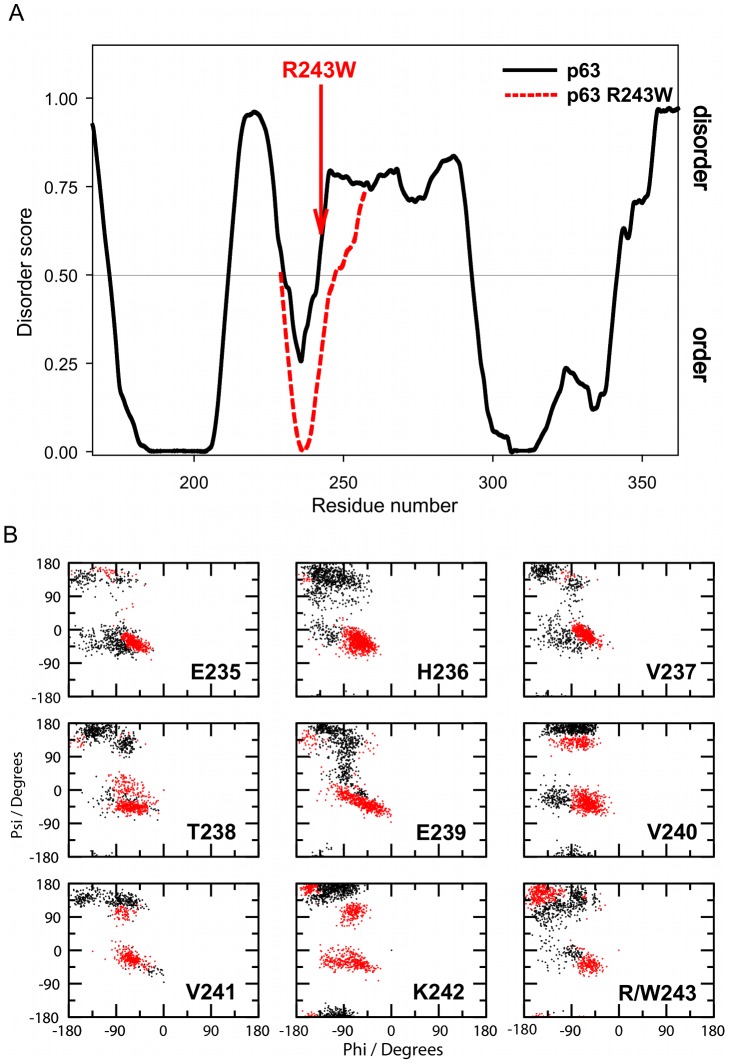
Disorder predictions and the effect of a R243W missense mutation in human protein p63. (A) PONDR VLXT disorder predictions for wild type and mutant p63. R243W mutation causes drop in the disorder score of 235–245 region. (B) Differential free energy weighted φ/ψ propensity plots for residues 235–243 obtained from AMD simulations performed on the wild-type and R243W mutant p63 DBD systems. The red dots represent those regions of the Ramachandran plot more heavily sampled by the mutant and the black dots represent those regions with greater propensity in the wild-type system.

We observed 670 mutations in UniProt predicted to cause D→O transitions, and 590 mutations predicted to cause O→D transitions (**Tables S10** and **S11**). We note that the number of such examples would be higher if extensively studied proteins with an excessively large number of mutations (such as p53, androgen receptor, etc.) were included in the analysis (**Materials and Methods** and **Figure S8**). In addition to disease mutations mapped to predicted disordered regions, we elsewhere summarized D→O disease mutations found in the experimentally ascertained disordered regions from the DisProt database [59]. Below, we show an example of a protein carrying predicted D→O disease mutation (**Figure 6**).

Tumor protein p63 (TP63) is a transcription factor involved in development and morphogenesis of epithelial tissues [60], [61]. The sequence, structure and domain organization of p63 are highly similar to tumor suppressor protein p53, with the exception of two additional domains at p63 C-terminus, which are alternatively spliced in some p63 isoforms. More than 30 distinct missense mutations have been identified in p63 and associated with several malformation genetic syndromes such as ectrodactyly ectodermal dysplasia-cleft syndrome 3 (EEC3, MIM: 604292), split hand/foot malformation-4 (SHFM4, MIM: 605289), and nonsyndromic cleft lip (NSCL, MIM: 129400). Most of the mutations that cause EEC3 occur within the DNA-binding domain of p63 [62]. One of these mutations, R243W, is predicted to cause a D→O transition, shown in **Figure 6A** as a sharp drop in disorder score of the 235–245 region (red dotted line) after R243 has been *in silico* mutated to W. Since R243 is not directly involved in binding to DNA, the mutations affecting this residue are predicted to destabilize the protein as a result of hydrogen bond loss and overpacking [63].

DNA-binding domains of transcription factors tend to be predicted as fully or partially disordered [64], [65], and binding to DNA typically induces a D→O transition [66]. In agreement with these observations, only a single NMR structure of p63 DBD without DNA (PBD: 2RMN) is available, while all X-ray structures of p63 DBD found in PDB (PDB: 3US0, 3US1, 3US2, 3QYM and 3QYN) have been crystallized in complex with DNA. Residue R243 is located in the modeled turn region of the NMR structure, adjacent to a short α-helix. We investigated the effects of the R243W mutation on p63 DBD conformation using an extensive set of accelerated molecular dynamics (AMD) simulations [67], [68] on both the wild-type p63 (wt-p63) and its R243W mutant.

AMD is an efficient and versatile enhanced conformational space sampling algorithm that has previously been successfully applied to the study of the conformational behavior of IDPs [69], [70]. A comparative analysis of a series of AMD trajectories for wt-p63 and its R243W mutant revealed no significant differences in the global structural dynamics of the p63 DBD. However, marked differences in the conformational behavior of residues adjacent to R243W were observed (**Figure 6B**). The introduction of R243W mutation caused a significant increase in the free energy weighted φ/ψ propensity of the α-helical/frustrated α-helical conformation of these residues, resulting α-helical population statistics of 70–90% and 30%–50% in the R243W mutant for residues 236–240 and 241–243 respectively, compared to 20%–60% and 20–25% in the wild-type system (**Table S12**). The formation of an ostensibly exclusive (frustrated) α-helical coil in this region in the presence of the R243W mutation is fully consistent with the predicted D→O transition (**Figure 6A**).

It is interesting to note that in both the experimental NMR structure and the AMD simulations for wt-p63 the side-chain of R243 forms a strong salt-bridge with E252. One may postulate that in the wild-type system the strong electrostatic interaction between R243 and E252 introduces tensile stress in the extended loop region K232-R243, which exhibits conformational exchange on slow time-scales between local extended β-sheet/PPII and α-helical constructs. By contrast, the introduction of the R243W mutation removes the tensile strain from the loop facilitating the formation of a stable α-helix.

## Discussion

The widely accepted structure-centric view of deleterious mutations asserts that a disease may be caused by mutations disrupting protein activity, stability, oligomerization and other structure-based properties. Here, we further extend this concept by introducing a disorder-centric view of disease mutations, according to which a disease may arise due to a disruption of the disorder-based protein properties [59]. We have demonstrated that **a substantial fraction of disease-associated mutations are located within the intrinsically disordered protein regions, and that disease mutations in IDRs have a significant functional impact** despite the fact that IDRs lack fixed structure and have fewer evolutionary constraints than ORs [32]. The analysis of mutations in IDRs shows that disorder-to-order transition mutations may be especially relevant to disease due to their enrichment compared to control datasets. In addition, our analysis suggests that several types of disease mutations may have particularly critical impact on disordered structure.

There are many ways in which mutations in IDR may increase disease risk or cause a disease. For example, D→O mutations have a potential to alter interactions with DNA, RNA, proteins or ligands. Both, our results and those of a recent study by Dan *et al.* [71], which examined transitions between disorder and secondary structure in proteins with solved 3D structures, converged on the observation that disorder-to-order (i.e. disorder-to-secondary structure in [71]) transitions are significantly enriched in DNA binding proteins. In addition, mutations in IDR could influence posttranslational modifications, assembly of macromolecular complexes, as well as signaling and regulatory processes that depend on disorder. Adding support to this hypothesis is an observation that disease mutations often disrupt anchoring of flexible loops of the catalytic domains in protein kinases, and that mutated residues are frequently involved in substrate binding and regulation [72]. This also suggests a potential downstream effect of mutations in IDR via dysregulation of cellular pathways which could lead to disease [59].

Our results show that across all three datasets, mutations in IDR are more likely to cause a predicted D→O transition than mutations in ORs are to cause a predicted O→D transition (**Table 2**). This is in agreement with a recent study by Schaefer *et al.* [73], which showed that disordered regions are more sensitive to mutations than protein regions with defined secondary structure, with a caveat that “order” and “helix or strand” cannot be fully equated. Despite a significant enrichment of D→O mutations in disease, the majority of disease mutations in IDR do not result in a disorder-to-order transition (as defined in this paper) but they nonetheless sufficiently disrupt the disordered conformation to affect disorder-mediated functions. It is likely however that many other mutations that do not reach the disorder-to-order transition threshold may still disrupt the structure and consequently function of the disordered regions.

Our findings have wide implications for large genome sequencing projects that aim to provide a better understanding of human genetic variation and its relevance to complex diseases [1]. Because the sheer volume of the observed variants precludes systematic functional follow-up studies on each one individually, newly identified SNVs are short-listed and prioritized using predictors of the functional impact of SNVs, such as SIFT, PolyPhen-2 and others [2]. The majority of the currently used predictors are structure- and/or conservation-based, and therefore less accurate on variants in unstructured and non-conserved protein regions. Disorder predictions could be either integrated into current approaches, or new approaches, which analyze the features of mutations in ORs and IDRs separately, could be developed. In addition, in this study we demonstrate that specific types of mutations (such as R→W, R→C, etc.) account for almost one half of all D→O transitions (**Figure 4**). This additional information may be important to include as a training feature when developing new predictors for the effects of D→O SNVs.

A broader issue raised by our results is that caution should be exercised when interpreting the relationship between structure, function and conservation. A study by Yue and Moult found that human disease-relevant mutations in some cases could correspond to the wild-type variants in the mouse [11]. Compensatory mutations [74] illustrate that function cannot be fully equated with the “first order conservation”, and that sometimes co-evolution of amino acids constrained by protein structure necessitates looking into the “second order conservation” between pairs of residues. Our results are consistent with the fact that IDRs are less conserved at any individual position, but rather show a conservation of disorder propensity within a region [75], with D***→***O transition mutations – detrimental to conservation of disorder – being particularly enriched in disease.

Choosing an appropriate control for the analysis of disease mutations is an issue which deserves close attention [76]. One of our control datasets, polymorphisms from UniProt (Poly), is likely to contain a fraction of as yet unannotated disease mutations, because it was assembled by translating missense single nucleotide variants (currently without any disease associations) into single amino acid changes [76], [77]. This is further supported by the predictive result that between 20% [7] and 25% [11] of non-synonymous SNPs are likely to be associated with diseases. Nonetheless, Poly controls for an important previously identified confounder: because disease missense mutations are translations of a single nucleotide variation within a DNA codon, a genetically appropriate control has to be analogously constrained by the genetic code, that is, assembled from amino acid changes which are translations of functionally neutral SNVs [57], [76]. In the protein space, another concern is that length distribution and amino acid compositions of proteins from DM and Poly datasets differ, which may influence their baseline biochemical properties, including disorder content (**Figure S1**). In order to address this potential confounder, the second control (NES) was generated starting with the sequences of proteins from the DM dataset. The downside of this approach is that the set of disease mutations spans within-population differences, while changes in the orthologs span larger, inter-species distances. In practice, this means that in DM and Poly the mutation probability matrix is dominated by the effects of the genetic code, while in DM and NES it is dominated by effects of physico-chemical similarity between amino acids. Nonetheless, variants fixed between species are likely to be non-deleterious (even though about 9% of interspecies substitutions have been estimated to be damaging [7]), and therefore they provide a useful additional control that takes into account sequence conservation. In the light of advantages and shortcomings of different control datasets, it is reassuring to see that when using either Poly or NES, protein disorder-related properties (**Tables 1** and **2**) and WT-to-mutant amino acid changes (**Figure 4**) are consistent and independent of the control dataset used. In addition, the preponderance of annotated mutations within OR might show some degree of ascertainment bias since some disease mutations were annotated as “disease” because they were mapped to protein structured domains. We hypothesize that an unbiased sample would contain a higher proportion of disease mutations that map to IDRs.

In summary, our results refine the traditional structure-centric view of disease mutations, and suggest new avenues for research in the area of protein disorder. With the recent explosion of exome and whole genome sequencing efforts, interpretation of the identified variants will require highly accurate predictors for the functional impact of SNVs in order to make reliable conclusions about their health risks. Our results offer help in narrowing down the gamut of disease mutations that dramatically influence protein structure and disorder. We hope that it will also facilitate predictions of the influence of mutations on protein function, which is currently a formidable task. The importance of mutations in disordered regions should not be overlooked in an attempt to construct better predictors.

## Materials and Methods

### Datasets

A list of single amino acid substitutions annotated with the keyword “disease” was extracted from the UniProt/SwissProt database [77]. This manually curated catalog contains missense mutations associated with both Mendelian and complex diseases, but no nonsense nor frame shift mutations, and no products of alternative splicing.

The initial set of mutations was filtered as follows: proteins that carry disease mutations and have ≥40% pairwise sequence identity were clustered using hierarchical clustering with single linkage, and one representative protein was selected at random from each cluster. We further removed four proteins with an unusually high number of annotated disease mutations (**Figure S8A**): tumor suppressor p53 (P04637), coagulation factor VIII (P00451), androgen receptor (P10275), and Stargardt disease protein (P78363). Taken together, these four proteins account for a total of 12.4% of all disease mutations found in the non-redundant set of proteins. All mutations from the removed proteins were discarded.

We assembled two control datasets: (1) annotated single amino acid polymorphisms from UniProt (Poly) [77] and (2) a set of pseudo-mutations based on amino acid variation in mammalian orthologous proteins (neutral evolutionary substitutions, NES). The first control dataset (Poly) was filtered analogously to disease mutations, and redundant proteins and titin (with unusually high number of polymorphisms) were removed (**Figure S8B**).

The second control dataset (NES) (**Figure S8C**) was constructed following the approach of Sunyaev *et al.* [78]. Proteins that carry disease mutations which also passed our filtering criteria were aligned by the use of multiple sequence alignment program MUSCLE [79] against their InParanoid [80] orthologs from 10 mammalian species (*P. troglodytes*, *P. pygmaeus abelii*, *M. musculus*, *M. mulatta*, *C. familiaris*, *E. caballus*, *R. norvegicus*, *C. porcellus*, *B. taurus*, and *M. domestica*), using the BLOSUM85 matrix. The set of neutral evolutionary substitutions (NES) was assembled from all single amino acid differences in orthologous proteins that had ≥95% sequence identity with the human disease protein. Finally, all annotated disease mutations were filtered out from the NES dataset. The numbers of proteins and mutations in the three datasets are summarized in **Table 1**.

### Disorder predictions

Protein disorder was predicted using VLXT [36], VSL2B [37] and IUPRED [38]. Disorder predictions were carried out on full length wild-type (WT) and mutated protein sequences, generated by changing only one residue at a time. Disorder score <0.5 signified predicted order and ≥0.5 signified predicted disorder. We defined the effect of a mutation as a disorder-to-order (D→O) transition if the prediction score for a residue to be mutated was ≥0.5 in the WT protein, and <0.5 after the mutation. Order-to-disorder (O→D) transitions were analogously defined. The enrichment/depletion trends for D→O and O→D transitions are consistent across all three predictors (**Tables S1** and **S3**).

As a second comparison of disorder predictors, we examined the distributions of the difference between disorder prediction scores on WT and mutated sequences, defined as Δps = ps(WT residue)−ps(mutated). The three predictors have different observed dynamic ranges for Δps: [−0.91, 0.85] for VLXT, [−0.34, 0.39] for VSL2B and [−0.28, 0.27] for IUPRED, consistent with the fact that VLXT is more sensitive to small changes in amino acid sequence. Distribution of Δps is more platykurtic in DM compared to Poly and NES for all three predictors (higher % of disease-associated mutations in the tails), indicating that disease mutations tend to cause stronger differences in prediction scores.

### Secondary structure predictions

Secondary structure was predicted from sequence using PHDsec [81]. We used only reliable predictions, defined as having both a “from” and “to” secondary structure assignment score ≥4. We note, however, that the trend was the same when all secondary structure predictions were used without thresholding on the reliability score.

### α-MoRF predictions

α-MoRFs were predicted from sequence using a two stage stacked prediction method [44]. The first stage identified potential α-MoRF regions from PONDR VLXT [36] predictions by scanning for short predicted ordered regions flanked by predicted disordered regions. The second stage classified potential α-MoRF regions as either α-MoRFs or non-α-MoRFs using a quadratic discrimination model [44]. Further details of α-MoRF predictions are provided in the **Supplementary Text S1**.

### Functional characterization

Residues were functionally annotated using the UniProt/SwissProt feature table (FT) at two levels of granularity, the FT keywords only (level 1) and concatenations of the FT keyword and description (level 2). Features marked as “Potential”, ”Probable” or “By similarity” were removed. The “Description” field was normalized by removing prefixes such as “For”, “Required for”, “Sufficient for”, “Essential for”, “Essential to”, “Important for”, “Critical for”, “Necessary for”, “Involved in”, “Mediates”, etc. Finally, all features that occurred <5 times in DM were removed. After this process, 22 level 1 and 782 level 2 features remained. We removed all disease keywords from this analysis, since they would be trivially enriched in the DM dataset.

### Molecular dynamics simulations

Standard classical and accelerated molecular dynamics simulations were performed on both wild-type and R243W p63 mutant using an in-house modified version of the AMBER-10 simulation suite [82]. The reader is referred to the Supplementary Information (**Supplementary Text S2**) for a description of the accelerated molecular dynamics method and computational details.

## Supporting Information

Figure S1 Histograms of the distribution of proteins in DM and Poly datasets with x% of residues predicted to be disordered by VLXT. The lower mode and shorter right tail of the DM distribution indicates that on average proteins carrying disease-associated mutations (DM) are less disordered than proteins carrying polymorphisms (Poly) (mean±SD 32.7±17.9% disorder *vs* 35.3±19.5%).
(TIF)

Figure S2 Summary of the effect of mutations in DM, Poly, NES on predicted molecular recognition features (α-MoRFs). Disease D→O transition mutations lead to a loss, while O→D transition mutations lead to a gain of predicted MoRFs, significantly more frequently than control mutations (marked with an asterisk, and reproduced in **Figure 2** of the main text).
(TIF)

Figure S3 Frequencies of mutated residues across all proteins (A, B); in ordered regions (C, D), and in disordered regions (E, F). In panel (A) frequencies of amino acids across whole proteins were normalized by frequencies in human proteins from UniProt; (C) frequencies in ORs normalized with frequencies from PDBS25 (sequences of proteins with solved crystal structures from PBD, filtered at 25% pairwise sequence identity); and (E) frequencies in IDR with frequencies in experimentally confirmed disordered regions from the DisProt database, as described in (Vacic *et al.*, 2007).
(TIF)

Figure S4 Frequencies of residues mutated into (A, B) across all proteins, (C, D) in ordered regions and in (E, F) disordered regions only. Normalization was performed as in **Figure S3** and (Vacic *et al.*, 2007).
(TIF)

Figure S5 Frequencies of mutations from (A,B) and into (C,D) arginine in DM, Poly and NES mutation datasets. (A) and (C) were normalized by the frequencies of amino acids from human proteins in UniProt, as described in (Vacic *et al.*, 2007).
(TIF)

Figure S6 Histogram of PolyPhen-2 scores for (A) disease mutations shows drop in sensitivity for mutations in IDRs and specifically for D→D mutations, while scores for (B) neutral polymorphisms and (C) neutral evolutionary substitutions show a drop in specificity for D→O mutations. High scores indicate deleterious mutations.
(TIF)

Figure S7 Histogram of SIFT scores for (A) disease mutations shows drop in sensitivity for mutations in IDRs and specifically for D→D mutations, while scores for (B) neutral polymorphisms and (C) neutral evolutionary substitutions show a drop in specificity for D→O mutations. Scores≤0.05 indicate damaging mutations.
(TIF)

Figure S8 Scatter plots of the number of mutations per protein against the rank of the protein for (A) DM, (B) Poly and (C) NES. Disease mutation (DM) plot (A) identifies four proteins which have an unusually high number of annotated disease mutations: tumor suppressor p53 (P04637), coagulation factor VIII (P00451), androgen receptor (P10275), and Stargardt disease protein (P78363). Taken together, these 4 proteins account for a total of 12.4% of all disease mutations, and were removed from subsequent analysis. The protein with most mutations in plot (B) is titin (Q8WZ42), the longest protein in the human proteome as annotated in UniProt, which has been removed from the Poly dataset.
(TIF)

Table S1 Disease mutations have higher frequencies in ordered regions independently of the choice of predictor (VLXT, VSL3B, and IUPred).
(XLS)

Table S2 Comparison of mutation rates in amino acid substitutions per residue (mean ± standard deviation) in disordered (IDR) and ordered (OR) regions in three studied datasets, disease mutations (DM), polymorphisms (Poly) and neutral evolutionary substitutions (NES). *P*-values were computed using Students t test.
(XLS)

Table S3 Disorder-to-order transition mutations are significantly enriched in DM independently of the choice of predictor. Order-to-disorder transition mutations are significantly depleted in disease when compared to NES but not when compared to Poly (after multiple testing correction). *P*-values were computed using Fisher's exact test.
(XLS)

Table S4 PHD secondary structure predictions (E strand, H helix, L loop) for disease mutations (DM), polymorphisms (Poly) and neutral evolutionary substitutions (NES) show an enrichment of predicted helices (H) and strands (E) in DM, and a corresponding depletion of loops (L).
(XLS)

Table S5 Changes in PHD secondary structure predictions (E strand, H helix, L loop) upon mutations in disease mutations (DM), polymorphisms (Poly) and neutral evolutionary substitutions (NES) dataset.
(XLS)

Table S6 Number of mutations in disease (DM), polymorphisms (Poly) and neutral substitutions (NES) datasets mapped to human instances of the eukaryotic linear motifs (ELM). Compared to controls, IDR disease mutations are enriched in ELM regions. D→O mutations in DM are significantly enriched in ELM regions compared to NES.
(XLS)

Table S7 Fold difference for Swiss Prot FT Level 1 features between DM and Poly (first two rows), and DM and NES (last three rows) for D→O and O→D transitions. Only features with Bonferroni-corrected *P*-values<0.05 in either D→O or O→D were included.
(XLS)

Table S8 Fold difference for Swiss Prot FT Level 2 features between DM and Poly (first five rows), and DM and NES (the remaining rows) for D→O and O→D transitions. Since no features in DM/Poly and only two features in DM/NES passed the Bonferroni-corrected *P*-value cutoff, all features with uncorrected *P*-value<0.01 in either D→O or O→D were included. Features in bold have significantly different fold difference between disease mutations in D→O or O→D.
(XLS)

Table S9 PolyPhen-2 and SIFT calls for all mutations in DM, Poly and NES show a drop in sensitivity for calling disease-associated mutations in IDRs (bold font in row DM IDR) and a drop in specificity for D→O (bold font in rows Poly D→O and NES D→O).
(XLS)

Table S10 670 disease mutations from UniProt predicted to result in a D→O transition.
(XLS)

Table S11 590 disease mutations from UniProt predicted to result in a O→D transition.
(XLS)

Table S12 Summary of the AMD simulations as secondary structure propensities in DNA-binding domain of tumor protein p63 in the wild-type p63 (Before mutation) and in the R243W mutant (After mutation). “Difference” displays the differences between the wildtype and the R243W mutant and demonstrates than upon the mutation propensity towards α-helical conformation increases leading to a decrease in entropy of the sampled populations for all but one residue (K242). Abbreviations are as follows: β, β-sheet (−180<φ<−100, ψ>120); ppII, poly-proline II (−100<φ<0, ψ>120); α, α-helix (−100<φ<0, −75<ψ<−25); Frust. α, “frustrated” α-helix (−159<φ<−100, −75<ψ<−25); Entropy, Shannon's entropy of the residue propensities.
(XLS)

Text S1 Details of α-MoRF predictions.
(DOC)

Text S2 Details of accelerated molecular dynamics (AMD) simulations carried out on the wild-type and R243W mutant of p63 DNA-binding domain.
(DOC)
