# Supplementary figures and images for: Disease-Associated Mutations Disrupt Functionally Important Regions of Intrinsic Protein Disorder
Source: PLoS Comput Biol. 2012 Oct 4;8(10):e1002709. doi: 10.1371/journal.pcbi.1002709 (PMC3464192; doi:10.1371/journal.pcbi.1002709)

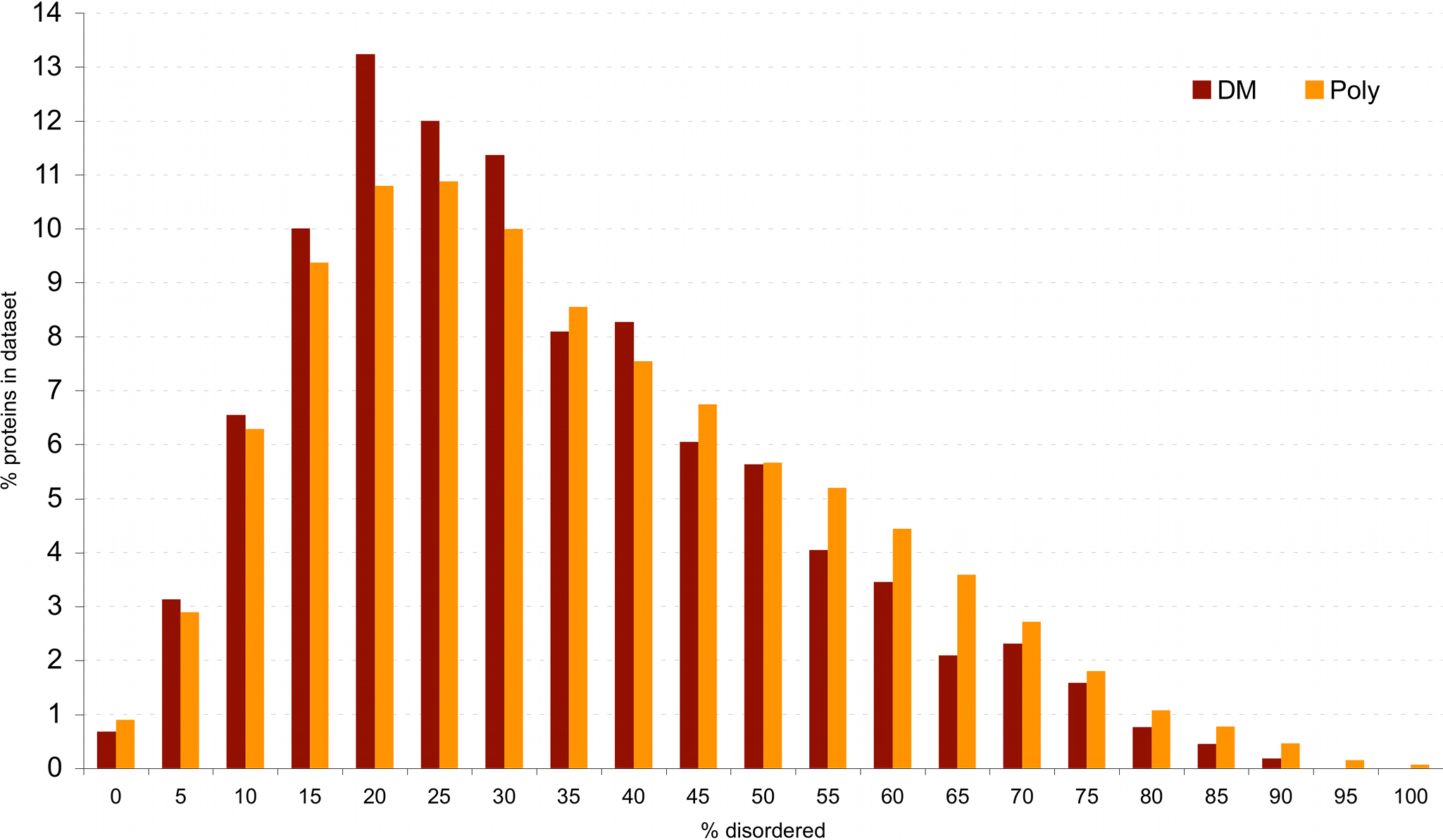

Supplement: Figure S1 — Histograms of the distribution of proteins in DM and Poly datasets with x% of residues predicted to be disordered by VLXT. The lower mode and shorter right tail of the DM distribution indicates that on average proteins carrying disease-associated mutations (DM) are less disordered than proteins carrying polymorphisms (Poly) (mean±SD 32.7±17.9% disorder vs 35.3±19.5%). (TIF) [file pcbi.1002709.s001.tif]

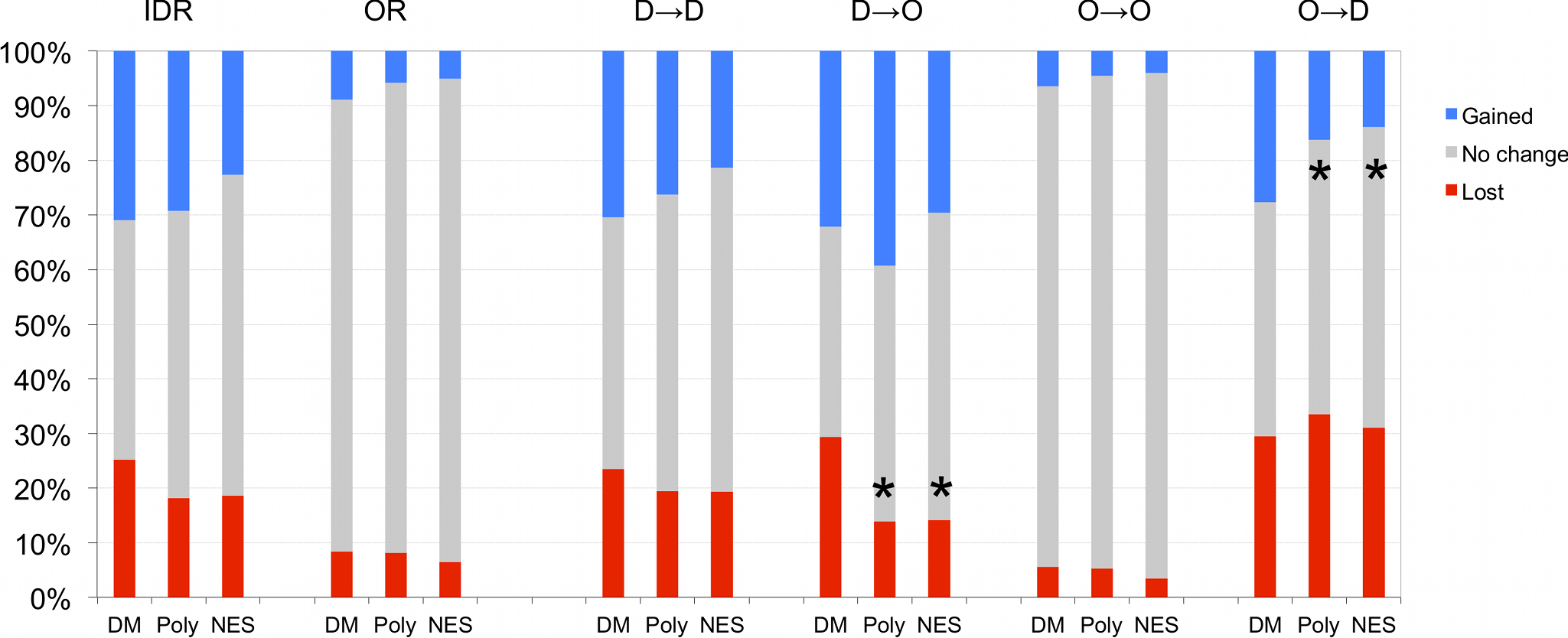

Supplement: Figure S2 — Summary of the effect of mutations in DM, Poly, NES on predicted molecular recognition features (α-MoRFs). Disease D→O transition mutations lead to a loss, while O→D transition mutations lead to a gain of predicted MoRFs, significantly more frequently than control mutations (marked with an asterisk, and reproduced in Figure 2 of the main text). (TIF) [file pcbi.1002709.s002.tif]

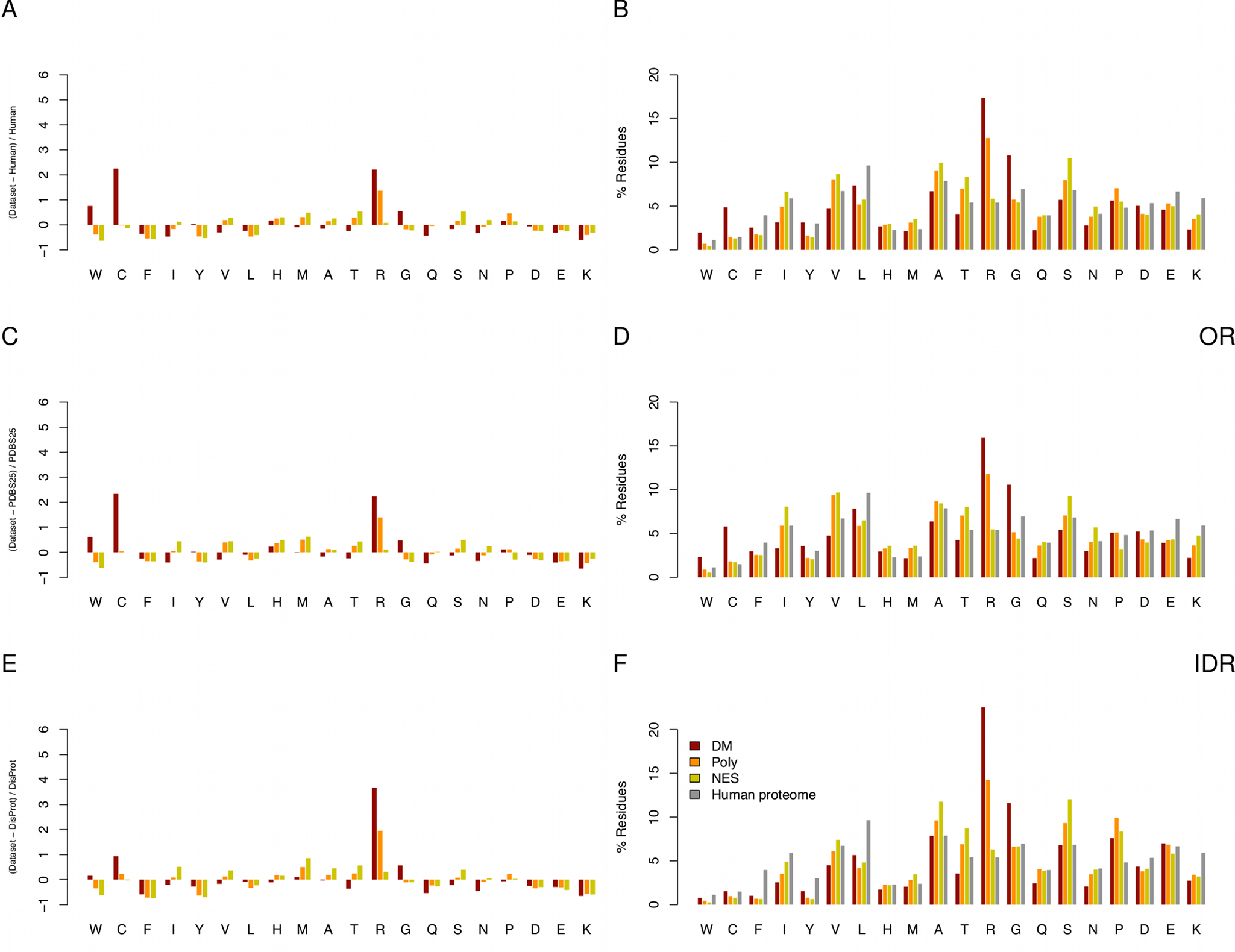

Supplement: Figure S3 — Frequencies of mutated residues across all proteins (A, B); in ordered regions (C, D), and in disordered regions (E, F). In panel (A) frequencies of amino acids across whole proteins were normalized by frequencies in human proteins from UniProt; (C) frequencies in ORs normalized with frequencies from PDBS25 (sequences of proteins with solved crystal structures from PBD, filtered at 25% pairwise sequence identity); and (E) frequencies in IDR with frequencies in experimentally confirmed disordered regions from the DisProt database, as described in (Vacic et al., 2007). (TIF) [file pcbi.1002709.s003.tif]

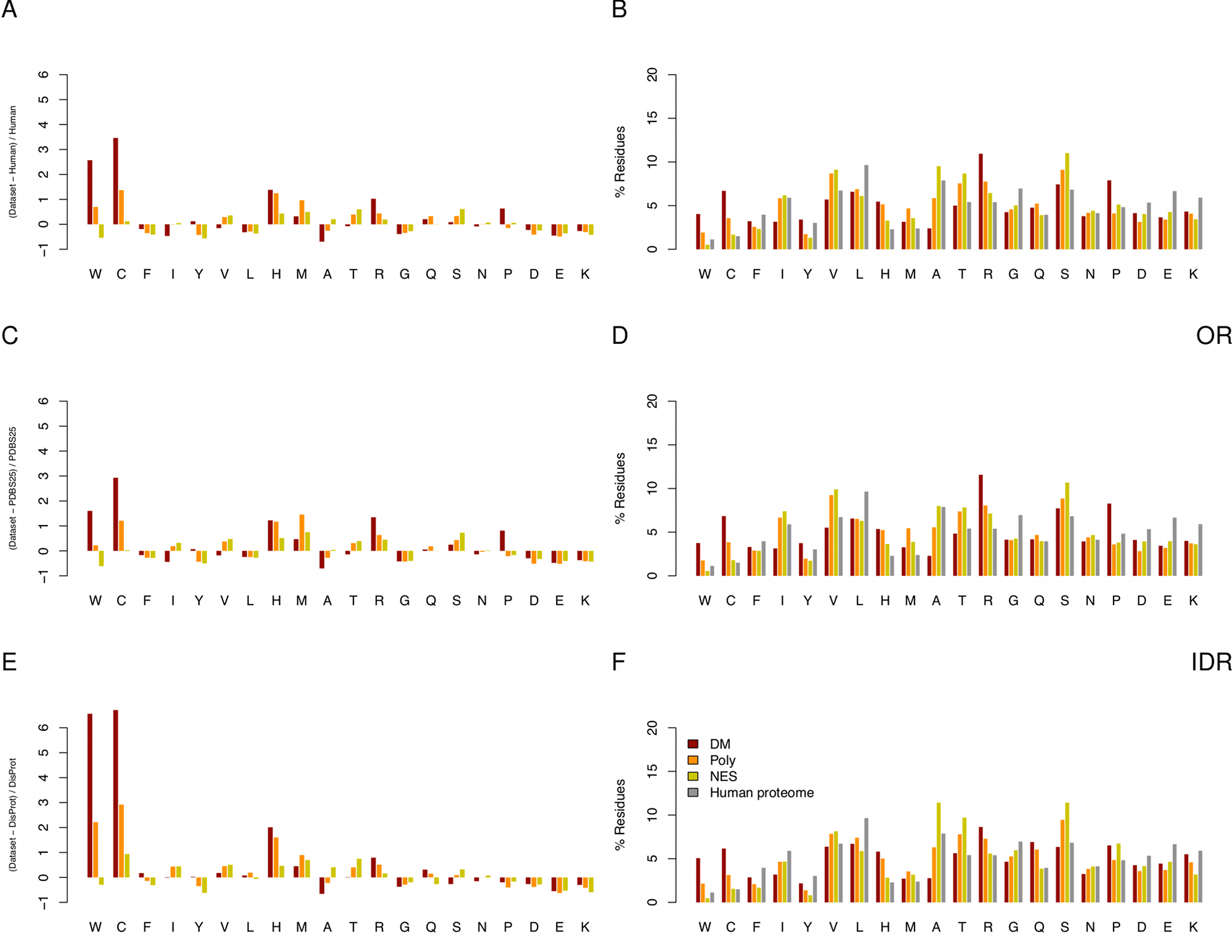

Supplement: Figure S4 — Frequencies of residues mutated into (A, B) across all proteins, (C, D) in ordered regions and in (E, F) disordered regions only. Normalization was performed as in Figure S3 and (Vacic et al., 2007). (TIF) [file pcbi.1002709.s004.tif]

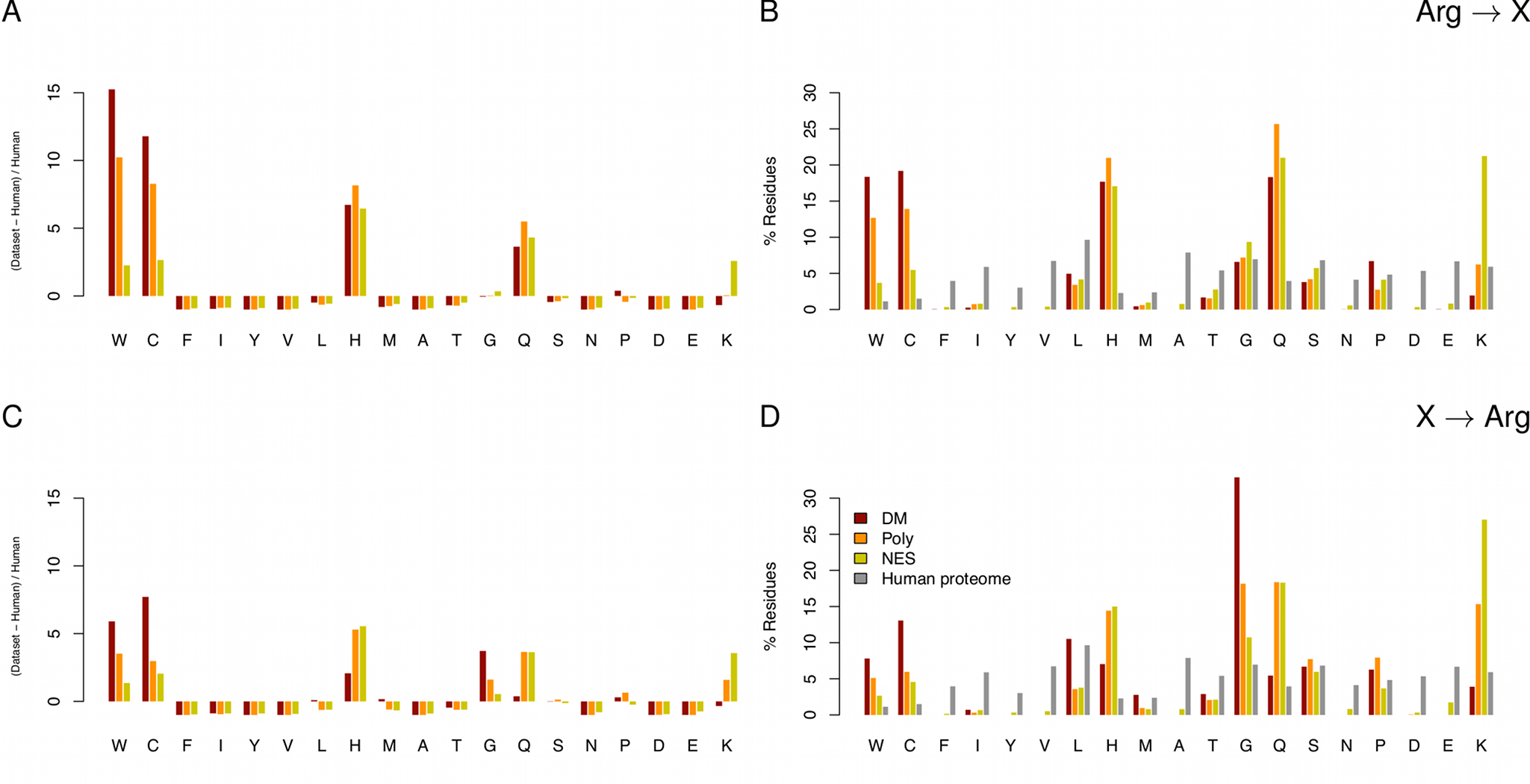

Supplement: Figure S5 — Frequencies of mutations from (A,B) and into (C,D) arginine in DM, Poly and NES mutation datasets. (A) and (C) were normalized by the frequencies of amino acids from human proteins in UniProt, as described in (Vacic et al., 2007). (TIF) [file pcbi.1002709.s005.tif]

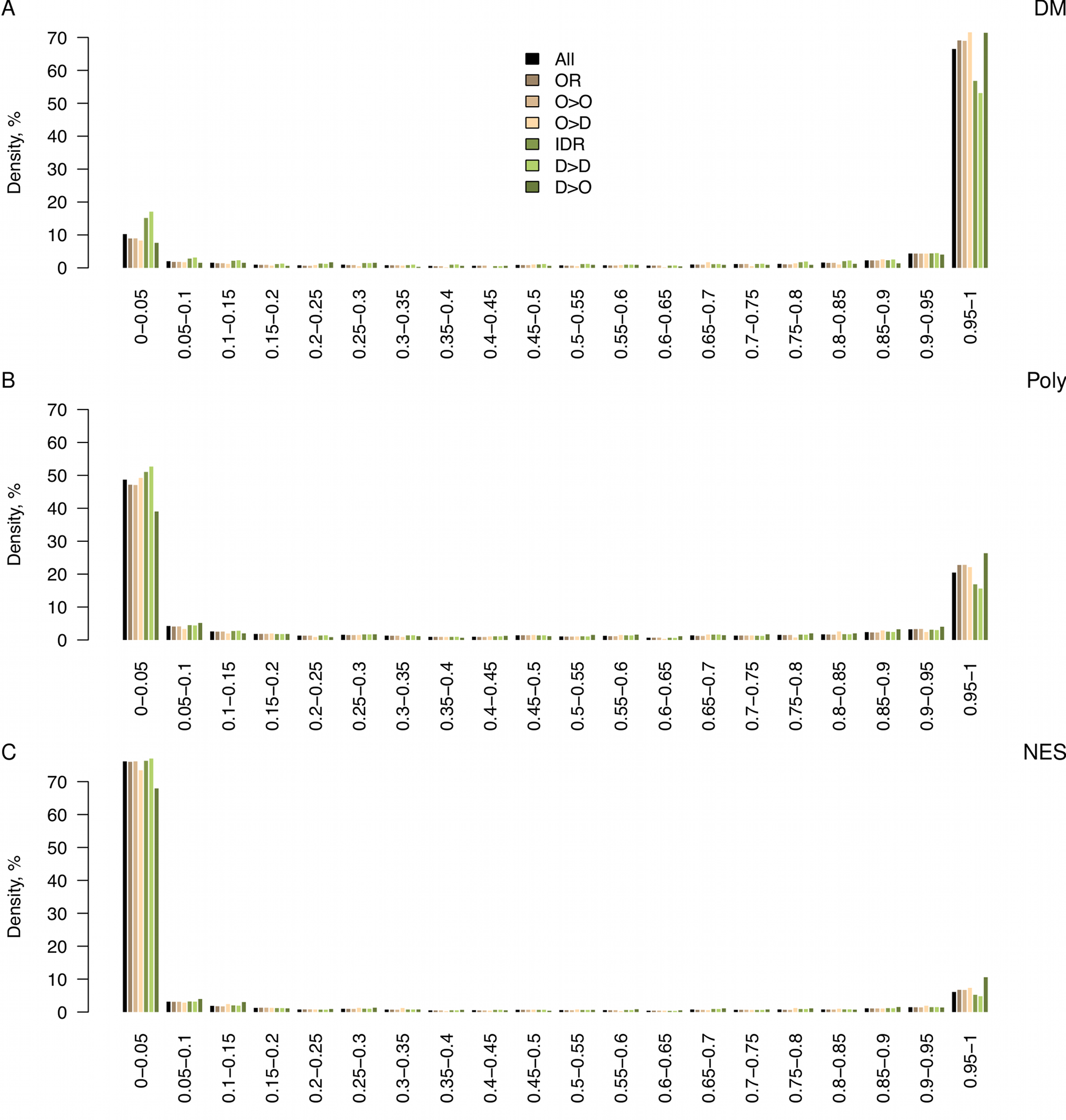

Supplement: Figure S6 — Histogram of PolyPhen-2 scores for (A) disease mutations shows drop in sensitivity for mutations in IDRs and specifically for D→D mutations, while scores for (B) neutral polymorphisms and (C) neutral evolutionary substitutions show a drop in specificity for D→O mutations. High scores indicate deleterious mutations. (TIF) [file pcbi.1002709.s006.tif]

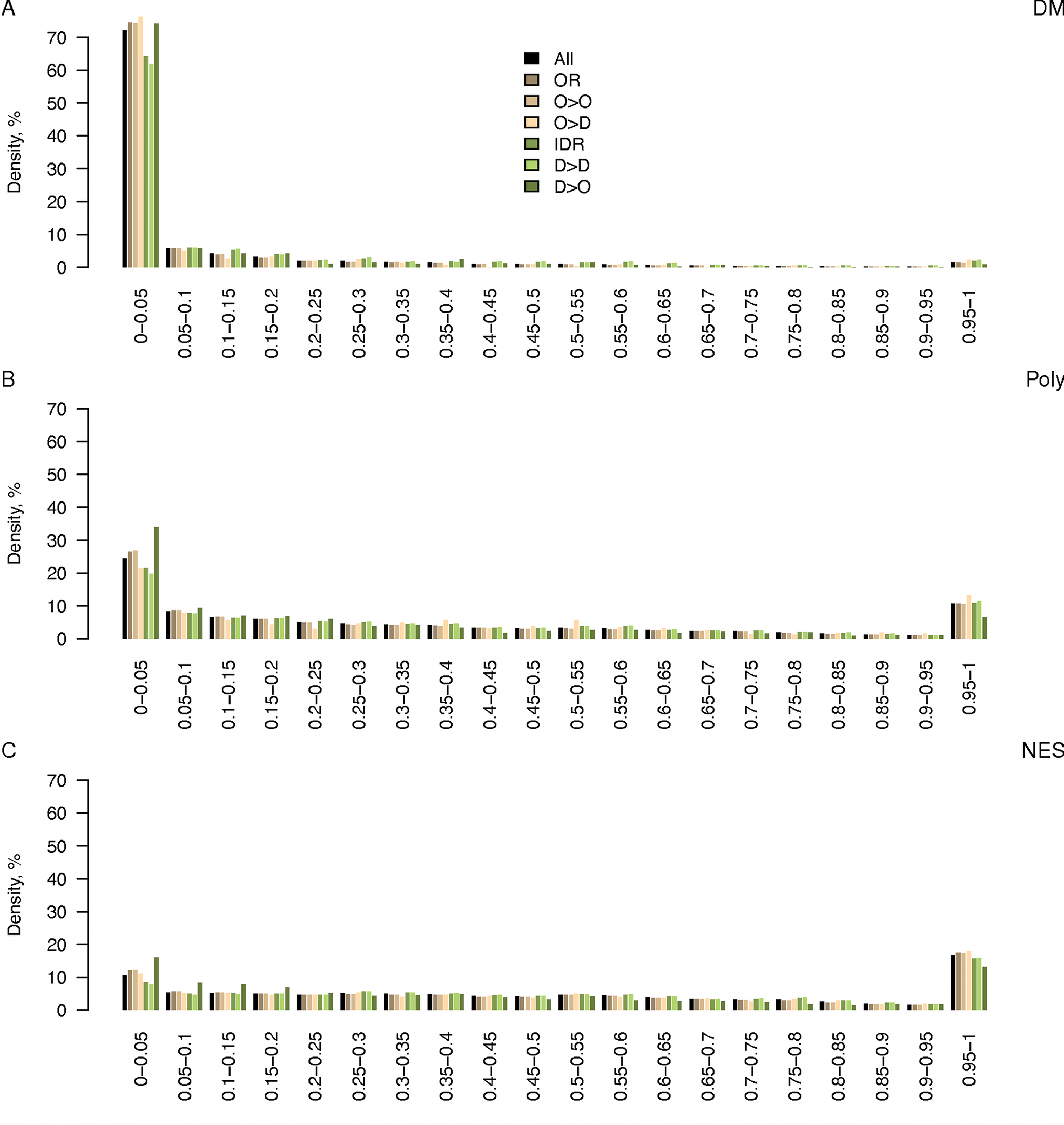

Supplement: Figure S7 — Histogram of SIFT scores for (A) disease mutations shows drop in sensitivity for mutations in IDRs and specifically for D→D mutations, while scores for (B) neutral polymorphisms and (C) neutral evolutionary substitutions show a drop in specificity for D→O mutations. Scores≤0.05 indicate damaging mutations. (TIF) [file pcbi.1002709.s007.tif]

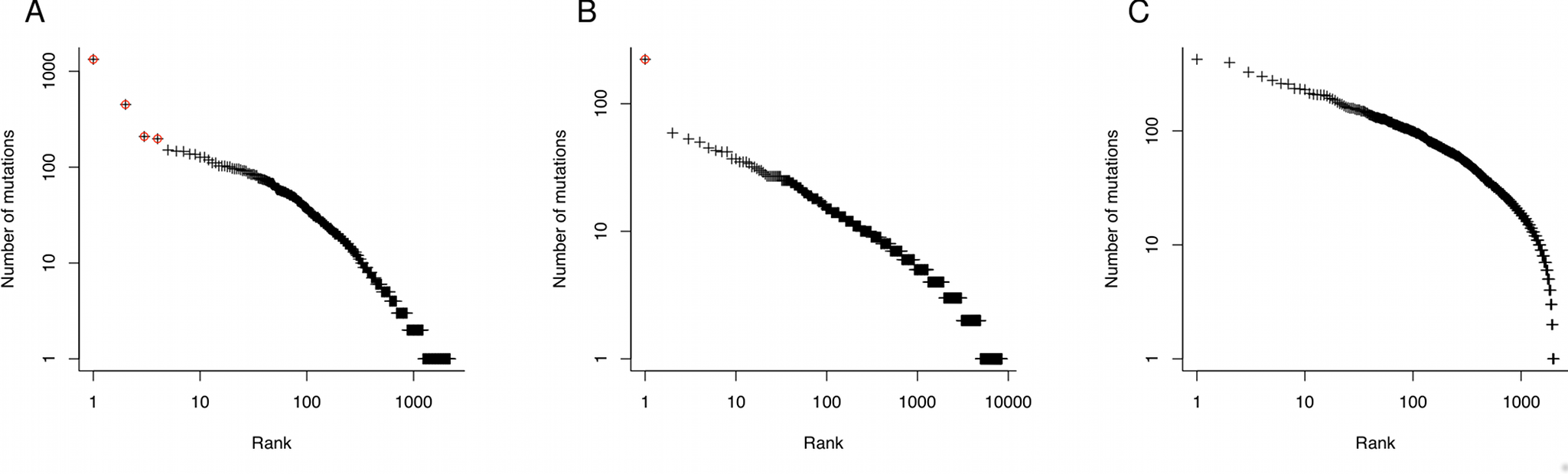

Supplement: Figure S8 — Scatter plots of the number of mutations per protein against the rank of the protein for (A) DM, (B) Poly and (C) NES. Disease mutation (DM) plot (A) identifies four proteins which have an unusually high number of annotated disease mutations: tumor suppressor p53 (P04637), coagulation factor VIII (P00451), androgen receptor (P10275), and Stargardt disease protein (P78363). Taken together, these 4 proteins account for a total of 12.4% of all disease mutations, and were removed from subsequent analysis. The protein with most mutations in plot (B) is titin (Q8WZ42), the longest protein in the human proteome as annotated in UniProt, which has been removed from the Poly dataset. (TIF) [file pcbi.1002709.s008.tif]
