# Supplementary material for: Disease-Associated Mutations Disrupt Functionally Important Regions of Intrinsic Protein Disorder
Source: PLoS Comput Biol. 2012 Oct 4;8(10):e1002709. doi: 10.1371/journal.pcbi.1002709 (PMC3464192; doi:10.1371/journal.pcbi.1002709)
Supplement: Text S1 — Details of α-MoRF predictions. (DOC) [file pcbi.1002709.s021.doc]

**Supplementary Information Text S1**

α**-MoRF predictions**

The predictor of α-MoRFs used here is based on the observation that binding residues within intrinsically disordered regions are often predicted to be ordered by PONDR VLXT . It was later found that α-MoRFs also follow this pattern, where the α-MoRF binding region corresponds to a short prediction of order within a long prediction of disorder . This behavior was used to construct a two-stage α-MoRF prediction method: first, all possible α-MoRF regions are identified from PONDR VLXT predictions, and second, possible α-MoRF regions are distinguished from spurious regions based on sequence properties .

For identification of possible α-MoRF regions, PONDR VLXT predictions were scanned to find short predictions of order within long disordered regions. Specifically, using an adjusted threshold of PONDR VLXT score of 0.6, potential α-MoRFs were defined as “ordered regions between 1 and 60 residues long, flanked by disordered regions, one of which must be at least 30 residues long”. The location of potential α-MoRF was finally defined as a 19 residues long segment, centered on the predicted ordered region.

The second stage of prediction was applied to distinguish between patterns corresponding to α-MoRF from those arising from other structural features. α-MoRF/non-α-MoRFs classification was performed with a quadratic discrimination model using 6 attributes of the potential α-MoRF region and flanking regions, including predicted secondary structure, predicted disorder, hydrophobic clusters , and hydrophobic moment among others. The classification model was fit to 14 α-MoRF examples combined with 863 potential α-MoRF regions identified in PDB Select 25 . No reliable estimate of the false negative rate of the stacked method is available, but the false positive rate on globular proteins is estimated at 6.0x10^-5^ false predictions per residue .

The α-MoRF prediction method has several implications for the current study, particularly with regard to perturbation of α-MoRF predictions by missense mutations. A mutation may effect the first step of α-MoRF prediction by either creating or destroying a possible α-MoRF region pattern, *e.g.* a D→O mutation within a long disordered region could create a new α-MoRF region pattern, or alternatively a O→D could completely remove an ordered region, thereby destroying a α-MoRF region pattern. For unperturbed patterns, the classification of a possible α-MoRF region may be changed due to the different properties of the mutated sequence.

Note that α-MoRF regions themselves may be predicted to be ordered or disordered. Typically, a threshold of 0.5 is applied to PONDR VLXT output, where predictions with the score ≥0.5 are classified as disordered, and predictions <0.5 are classified as ordered. This is the disorder prediction threshold used in this work to classify mutations. However, due to the 0.6 threshold used for the α-MoRF prediction, α-MoRFs may be predicted by PONDR VLXT to be entirely disordered, partially disordered, or entirely ordered. Therefore, D→O mutation can overlap with the predicted MoRF along with the O→D mutation, and they both could influence physico-chemical features used to predict MoRFs. With regard to biological effects, an O→D mutation may disrupt α-MoRF by reducing the capacity of a region to fold, and therefore its ability to recognize its target. Similarly a D→O mutation may result in a region with an increased propensity to fold, thereby creating a new α-MoRF, which may possibly recognize a novel target. It should be noted that α-MoRF prediction is in no way dependent on possible molecular partners: a prediction indicates that a region is consistent with the properties of a binding region, not that a region does bind to a particular partner.

**References**

1. Garner E, Romero P, Dunker AK, Brown C, Obradovic Z (1999) Predicting binding regions within disordered proteins. Genome Inform Ser Workshop Genome Inform 10: 41-50.

2. Oldfield CJ, Cheng Y, Cortese MS, Romero P, Uversky VN, et al. (2005) Coupled folding and binding with alpha-helix-forming molecular recognition elements. Biochemistry 44: 12454-12470.

3. Callebaut I, Labesse G, Durand P, Poupon A, Canard L, et al. (1997) Deciphering protein sequence information through hydrophobic cluster analysis (HCA): current status and perspectives. Cell Mol Life Sci 53: 621-645.

4. Eisenberg D, Weiss RM, Terwilliger TC (1982) The helical hydrophobic moment: a measure of the amphiphilicity of a helix. Nature 299: 371-374.

5. Hobohm U, Sander C (1994) Enlarged representative set of protein structures. Protein Sci 3: 522-524.
