# Supplementary material for: Disease-Associated Mutations Disrupt Functionally Important Regions of Intrinsic Protein Disorder
Source: PLoS Comput Biol. 2012 Oct 4;8(10):e1002709. doi: 10.1371/journal.pcbi.1002709 (PMC3464192; doi:10.1371/journal.pcbi.1002709)
Supplement: Text S2 — Details of accelerated molecular dynamics (AMD) simulations carried out on the wild-type and R243W mutant of p63 DNA-binding domain. (DOC) [file pcbi.1002709.s022.doc]

**Supplementary Information Text S2**

**Accelerated molecular dynamics simulations**

The details of the accelerated molecular dynamics (AMD) method have been discussed previously in the literature . The essential idea behind AMD is to define a reference, or boost energy, *E_b_*, which is fixed above the minimum of the potential energy sur­face (PES). At each step in the AMD simulation, if the potential energy of the system lies below *E_b_*, a continuous, non-negative bias is added to the actual potential. If the potential energy is greater than *E_b_*, it remains unaltered. Explicitly, the modified potential *V*(r)* is defined as:

$$V^{*}(r)=\left\{\begin{matrix} V(r), V(r)\geq E_{b} \\ V(r)+\Delta V(r), V(r)<E_{b} \end{matrix}\right.$$

This results in a raising and flattening of the potential energy landscape, as well as decreasing the magnitude of the energy barriers between low energy states. As a consequence, the escape rate from one low energy conformational state to another is enhanced, whilst the essential details of the underlying potential energy surface are maintained. The extent to which the potential energy surface is modified depends on the difference between the boost energy and the actual potential.

The energy modification or bias *ΔV(r)* is given by:

$$\Delta V(r)=\frac{\left(E_{b}-V(r)\right)^{2}}{\alpha+E_{b}-V(r)}$$

The extent of acceleration (*i.e.* how aggressively we enhance the conformational space sampling) can be increased by either increasing the boost energy, or decreasing the acceleration parameter α. During the course of the simulation, if the potential energy is modified, the forces on the atoms are recalculated for the modified potential. The use of the bias potential *ΔV(r)* ensures that the derivative of the modified potential will be continuous at points where *V(r)= E_b_.* It should be noted that if the boost energy is set too large, the modified potential energy surface becomes iso-energetic, resulting in a random walk through phase space.

One of the favorable characteristics of AMD is that it yields a canonical average of an observable, so that thermo­dynamic and other equilibrium properties of the system can be accurately determined. The corrected canonical ensemble average of the system is obtained by re-weighting each point in the configuration space on the modified potential by the strength of the Boltzmann factor of the bias energy, *exp[β ΔV (r)]* at that particular point. Whilst AMD represents a robust free energy sampling method, obtaining an accurate estimate of the time-scale of the observed conformational space sampling is extremely difficult as the application of the bias potential has a strong effect on the transmission coefficient. The only way to avoid this problem would be to set the boost energy below the entire transition state region, thereby preserving the transition-state theory formalism. However, considering that proteins possess a highly rugged and structured potential energy landscape, setting the boost energy below the entire transition state region on the protein PES would not allow acceleration over the larger energy barriers that separate the conformational substates.

In the present study, we have performed dual boost AMD simulations on both the wild type p63 (wt-p63) DNA binding domain and a single point mutation (R243W) of this system. In the framework of dual boost AMD, two acceleration potentials are applied simultaneously to the system: the first acceleration potential is applied to the torsional terms only, and a second, weaker acceleration potential is applied across all terms in the molecular mechanical force-ﬁeld. The torsional acceleration potential enhances local conformational transitions, whilst the weaker, background (total) acceleration potential acts to enhance the diffusion properties of the solvent molecules, thereby increasing the diffusive motions in the solute. Free energy (and hence population) statistics can be retrieved from dual boost AMD simulations by reweighting each point in the configurational space on the modified potential by *exp[β(ΔV ^d^(r_ti_)+ΔV ^T^ (r_ti_))]*, where *ΔV ^d^(r) and ΔV ^T^ (r)* represent the torsional and total background bias potential, respectively.

**Simulation details**

The NMR structure of the DNA binding domain of wt-p63 (PDB:2RMN, ) was placed in a periodically re­peating box with ~12,000 water molecules and an appropriate number of Cl- counter-ions, introduced to neutralize the simulation cell. Initially two standard classical MD simulations were performed. In each case, the system was brought to thermodynamic equilibrium at 300K, 1 bar pressure using a Langevin ther­mostat with a collision frequency of 2 ps^−1^ and a Berendsen weak-coupling pressure-stat. For the two MD simulations a different random seed generator for the Maxwellian distribution of atomic velocities was employed and, after standard energy minimization and equilibration procedures, a 5 ns production MD simulation was per­formed under periodic boundary conditions with a time-step of 2 fs. Bonds involving protons were constrained using the SHAKE algorithm. Electrostatic interactions were treated using the Particle Mesh Ewald (PME) method with a direct space sum limit of 10A. The ff99SB force-ﬁeld was used for the solute residues and the TIP4P water force-field was employed for the solvent molecules. These initial 5 ns MD simulations provided the starting point for the biased potential accelerated molecular dynamics simula­tions discussed below. An identical procedure was performed for the R243W mutant system, whereby initially residue R243 was manually changed to a W residue and any local steric clashes introduced by the presence of the W side-chain were removed during the energy minimization and equilibration runs.

For both the wild-type and R243W p63 DNA binding domain systems, two independent dual boost AMD simulations were performed for 5 · 10^7^ steps (the equivalent number of steps performed for 100 ns of standard classical MD using a time-step of 2 fs). For the torsional acceleration, the boost energy E_b_^d^ was defined such that *[E_b_^d^ −(V ^d^)]* = 930 kcal/mol, where *(V ^d^)* is the average dihedral angle energy obtained from the standard 5 ns classical MD sim­ulations and the acceleration parameter *α^d^* was set to 186 kcal/mol (one ﬁfth of the value of *[E_b_ −(V ^d^)]).* Similarly, for the total background acceleration, the boost energy *E_b_^T^* was defined such that *[E_b_^T^ −(V^T^ )]* was equal to 0.16 kcal/mol times the number of atoms in the simulation cell (NASC), and the total acceleration parameter *α^T^* was also set to 0.16 kcal/mol times NASC. The specific choice of these acceleration parameters was based on an analysis of previous successful AMD simulation studies (for more details see ). The physical conditions, force-fields and all other simulation parameters employed in the AMD simulations were identical to those described above for the standard 5 ns classical MD trajectories. All MD and AMD simulations were performed using an in-house modified version of the AMBER 10 code .

**Analysis of the conformational behavior of wt- and R243W mutant p63 DNA binding domain systems: Ex­tracting free energy weighted Ramachandran plots from the AMD simulations**

In order to identify differences in the local backbone *φ/ψ* conformational behavior of wt- and R243W p63 DNA binding domains, free energy weighted Ramachandran plots were calculated for each residue in the vicinity of the R243W mutation (specifically residues 235 to 243), which are depicted in **Figure 6B** in the manuscript. In order to perform this analysis, *φ/ψ* pairs were collected for each residue across the AMD simulations for both the wt­ and R243W mutant systems. For each residue, the Ramachandran space was divided into 10° by 10° bins, and each *φ/ψ* pair was allocated to its appropriate bin. Canonical Boltzmann distributions were obtained by multiplying each *φ/ψ* point by its Boltzmann weighting factor (see above) using the bias potential block averaging method to remove statistical noise errors. Free energy statistics were then calculated as *ΔG = −RT ln(N_i_/N_0_),* where *N_i_* is the population of each bin *_i_* and *N_0_* is the most populated bin. The population difference between the wt- and R243W mutant were calculated for each bin and a random number generator was used to obtain a representative sample of *φ/ψ* points that map out the differential population statistics.

**References**

1. Hamelberg D, Mongan J, McCammon JA (2004) Accelerated molecular dynamics: a promising and efficient simulation method for biomolecules. J Chem Phys 120: 11919-11929.

2. Hamelberg D, de Oliveira CA, McCammon JA (2007) Sampling of slow diffusive conformational transitions with accelerated molecular dynamics. J Chem Phys 127: 155102.

3. Markwick PR, McCammon JA (2011) Studying functional dynamics in bio-molecules using accelerated molecular dynamics. Phys Chem Chem Phys 13: 20053-20065.

4. Furrer J, Enthart A, Kuhlewein A, Dehner A, Klein C, et al. (2003) Backbone 1H, 13C and 15N resonance assignments for the 25.8 kDa DNA binding domain of the human p63 protein. J Biomol NMR 26: 377-378.

5. Cheatham TE, 3rd, Miller JL, Fox T, Darden TA, Kollman PA (1995) Molecular Dynamics Simulations on Solvated Biomolecular Systems: The Particle Mesh Ewald Method Leads to Stable Trajectories of DNA, RNA, and Proteins. J Am Chem Soc 117: 4193–4194.

6. Hornak V, Abel R, Okur A, Strockbine B, Roitberg A, et al. (2006) Comparison of multiple Amber force fields and development of improved protein backbone parameters. Proteins 65: 712-725.

7. Case DA, Darden TA, Cheatham TE, Simmerling CL, Wang J, et al. (2008) AMBER 10. University of California, San Franciasco.
